# Relationship of Height to Site‐Specific Fracture Risk in Postmenopausal Women

**DOI:** 10.1002/jbmr.2742

**Published:** 2015-12-06

**Authors:** Miranda EG Armstrong, Oksana Kirichek, Benjamin J Cairns, Jane Green, Gillian K Reeves, Valerie Beral for the Million Women Study Collaborators

**Affiliations:** ^1^ Cancer Epidemiology UnitUniversity of OxfordOxfordUK

**Keywords:** MILLION WOMEN STUDY, FRACTURE, HEIGHT, PROSPECTIVE STUDIES, POSTMENOPAUSAL

## Abstract

Height has been associated with increased risk of fracture of the neck of femur. However, information on the association of height with fractures at other sites is limited and conflicting. A total of 796,081 postmenopausal women, who reported on health and lifestyle factors including a history of previous fractures and osteoporosis, were followed for 8 years for incident fracture at various sites by record linkage to National Health Service hospital admission data. Adjusted relative risks of fracture at different sites per 10‐cm increase in height were estimated using Cox regression. Numbers with site‐specific fractures were: humerus (3036 cases), radius and/or ulna (1775), wrist (9684), neck of femur (5734), femur (not neck) (713), patella (649), tibia and/or fibula (1811), ankle (5523), and clavicle/spine/rib (2174). The risk of fracture of the neck of femur increased with increasing height (relative risk [RR] = 1.48 per 10‐cm increase, 99% confidence interval [CI] 1.39–1.57) and the proportional increase in risk was significantly greater than for all other fracture sites (*p*_heterogeneity_ < 0.001). For the other sites, fracture risk also increased with height (RR = 1.15 per 10 cm, CI 1.12–1.18), but there was only very weak evidence of a possible difference in risk between the sites (*p*_heterogeneity_ = 0.03). In conclusion, taller women are at increased risk of fracture, especially of the neck of femur. © 2015 The Authors. Journal of Bone and Mineral Research published by Wiley Periodicals, Inc. on behalf of American Society for Bone and Mineral Research (ASBMR).

## Introduction

Height may influence fracture risk through various biomechanical mechanisms, including differences in impact forces after a fall;^1^, ^2^, ^3^ moment arm length;^4^ geometric features of bone, such as hip axis length;^4^, ^5^, ^6^, ^7^ and bone structure, especially in long bones.^8^, ^9^ Although an increased risk of neck of femur fracture has been associated with increasing height,^10^, ^11^, ^12^ there is limited information on the relationship between height and fracture risk at other specific sites. Studies that have considered associations between height and sites other than neck of femur have often been small, with limited power to detect differences,^13^, ^14^, ^15^, ^16^, ^17^, ^18^, ^19^, ^20^ so there is a lack of consensus on the associations of height and site‐specific fracture risk.

Therefore, we examined the relationship of height with risk of hospital admission for fracture at nine specific sites among postmenopausal women in the Million Women Study, a large prospective study of women in the UK.

## Materials and Methods

### Participants and data

From 1996 to 2001, 1.3 million middle‐aged women in the UK were recruited into the Million Women Study through National Health Service (NHS) breast‐screening clinics located in England and Scotland. Further details of the cohort and study recruitment methods have been described elsewhere.^21^ Baseline for these analyses was the resurvey questionnaire administered about 3 years after recruitment into the study, in which women answered questions on health and lifestyle factors and also on a history of osteoporosis and of past fracture and falls, which are important risk factors for fracture. Study questionnaires and further details of the data and access policies can be viewed on the website (www.millionwomenstudy.org). Permission to conduct the study was provided by the Oxford and Anglia Multi‐Centre Research Ethics Committee.

Each woman was registered with the NHS and had a unique NHS identification number. This NHS number, in conjunction with other personal information, was used to link each woman to her cause‐specific information on NHS hospital admissions databases (including inpatient, ie, overnight, and day‐case, ie, not overnight, admissions): in England, Hospital Episodes Statistics,^22^ and in Scotland, Scottish morbidity records.^23^ Details on date of hospital admission and discharge and on diagnoses and procedures associated with each admission were recorded by those providing the hospital episodes data, coded to the World Health Organization's International Classification of Diseases, 10th revision (ICD‐10)^24^ for diagnoses. Follow‐up is virtually complete, with only 1% of the study population lost to follow‐up.

Our main endpoints were hospital admissions for incident fracture. Incident fractures at nine sites, identified according to ICD‐10 coding, were defined as the first primary or secondary diagnosis of fracture of humerus (S42.2–S42.4), radius and ulna (S52.0–S52.4, S52.7), wrist (S52.5–S52.6, S62.0–S62.1, S62.8), neck of femur (S72.0–S72.2), femur (not neck, S72.3–S72.4), patella (S82.0), tibia and fibula (S82.1–S82.2, S82.4), and ankle (S82.3, S82.5–S82.6, S82.8). Clavicle (S420), rib (S22.3–S22.5), and vertebral (S12.0–S12.2, S12.7, S22.0–S22.1, S32.0–S32.2) fractures were combined into a group of other main fracture sites. The group of all incident fractures was defined as incident fractures occurring at any of the above sites. Women with a history of prior fracture were defined as those with an incident fracture occurring before study baseline, which included the ICD‐10 codes mentioned above or any of the following ICD‐10 fracture codes: S02, S12, S22, S32, S42, S52, S62, S72, S82, S92, T02, T08, T10, T12, T14.2, M48.4, M80, and M84.3. Women self‐reporting a fracture at study baseline were also assumed to have a history of prior fracture. Women either reporting osteoporosis at study baseline or with an indication of osteoporosis in their hospital records before study baseline were assumed to have a history of osteoporosis.

### Body size

At recruitment into the study, women reported their height and weight in imperial units (feet and inches, and stones and pounds, respectively). These were converted to metric units (to the nearest 1 cm and 0.1 kg, respectively) and used to calculate body mass index (BMI; weight in kilograms divided by the square of height in meters). The height and weight of 2772 randomly selected study participants were measured a decade after recruitment and found to be strongly correlated with self‐reported heights and weights (height correlation coefficient = 0.88; weight correlation coefficient = 0.88). The majority of our analyses classified women in terms of self‐reported height categories (<155, 155.0–159.9, 160.0–164.9, 165.0–169.9, ≥170 cm), with mean measured height calculated within each category to adjust for the effects of reporting errors.^25^

### Statistical analysis

All analyses were conducted using the Stata, version 13, statistical package (StataCorp, College Station, TX, USA).^26^ Analyses were restricted to postmenopausal women. Because 96% of women in this cohort with a known age at natural menopause reported being postmenopausal by 55 years, women who were premenopausal, perimenopausal, or of unknown menopausal status at recruitment were assumed to be postmenopausal after they reached this age. Women were excluded from the analyses if they had a diagnosis of cancer or reported a stroke before study baseline. These exclusions were important because of the possible influence of these conditions on subsequent weight, physical activity, bone mineral density, and the propensity to fall.^27^, ^28^, ^29^

For women in Scotland, hospital admission data were available from January 1, 1981, until December 31, 2008. For women in England, hospital admission data were available from April 1, 1997, until March 31, 2011. Person‐years were computed from the date when the analysis baseline questionnaire was completed to whichever came first of the date of any of nine fracture sites of interest, date of death, date of emigration, or the end of follow‐up. When more than one incident fracture was reported during a single hospital admission, each fracture was included in the individual site analyses. Therefore, the total number of fractures across all sites may exceed the number of subjects with fracture. However, for all the analyses for risk of “any” fracture, each woman with multiple fractures was counted only once.

Cox regression models with attained age as the underlying time variable were used. Analyses were stratified by recruitment region (10 regions) and adjusted for socioeconomic status (quintiles), BMI (ie, <20, 20.0–22.5, 22.5–25.0, 25.0–27.5, 27.5–30.0, ≥30.0 kg/m^2^), strenuous physical activity (ie, no strenuous activity, up to 1 hour per week, more than 1 hour per week), smoking status (ie, current <15 cigarettes per day, current 15+ cigarettes per day, prior, never), alcohol consumption (ie, 0, 1–2, 3–6, 7–14, ≥15 drinks per week), use of menopausal hormones (ie, never, prior, current), history of prior fracture (either self‐reported or from hospital admission data: yes, no), history of osteoporosis (either self‐reported or from hospital admission data: yes, no), and diabetes (self‐reported: yes, no). Information on all variables were those provided at study baseline, except for height, parity, and socioeconomic status, which were provided at recruitment. To preserve sample size, records containing missing data or where the question was unasked (generally <2% for each variable) were retained, with the missing or unasked values coded as additional categories. A graphical assessment showed that the proportional hazards assumption was not violated by any of the models.

Relative risks (RRs) per 10‐cm increase in height were calculated as a trend across the six height‐category means using the mean measured height within each category of self‐reported height.^25^ To allow for valid comparisons between any two groups, the RRs were treated as group‐specific risks (also known as floating absolute risks) when more than two categories were used for risk comparisons.^30^ These risks were given as RRs followed by their corresponding group‐specific confidence interval (gsCI), allowing comparisons to be made between any two categories, even when neither is the reference category. When two specific categories were compared, conventional confidence intervals were used. We used 99% confidence intervals because of the many statistical tests done.

To assess heterogeneity of risks of fracture by height between different fracture sites, we used a competing risks Cox regression model. We fitted the proportional hazards model for the different fractures jointly, duplicating data for each type of fracture, applying censoring to the duplicated data, and including an interaction between failure type and the covariates in the model.^31^ We then used a likelihood ratio test to compare the model with a separate effect of height on each endpoint and the model with the same effect of height on each endpoint.

We assessed whether risks of fracture per 10‐cm increase in height differed between women by year of birth, deprivation level, smoking status, alcohol consumption, BMI, participation in strenuous physical activity, age at menarche, parity, use of hormone therapy, diabetic status, history of prior fracture, or history of osteoporosis.

We also conducted two sensitivity analyses, one excluding the first 2 years of follow‐up for all women and one excluding women with missing adjustment variables.

## Results

In total, 796,081 postmenopausal women were followed for incident fracture at various sites for an average of 8.4 years per woman (7 million person‐years in total). During this period, there were 28,431 hospital admissions (either day‐case or overnight stay) for relevant incident fractures. The numbers of women with a fracture at each specific site were: humerus (3036), radius and/or ulna (1775), wrist (9684), neck of femur (5734), femur (not neck) (713), patella (649), tibia and/or fibula (1811), ankle (5523), or clavicle/spine/rib (2174).

Baseline characteristics of the study population are described in Table 1, according to five categories of height. When compared with shorter women, taller women tended to have a lower BMI, smoke less, and consume a greater amount of alcohol. A smaller proportion of taller women reported prior osteoporosis, having diabetes, and being physically inactive. One‐fifth of women had reported having had one or more falls in the previous year, but this proportion did not differ much with height.

**Table 1:** Baseline Characteristics of Postmenopausal Women in the Million Women Study by Height and Follow‐up for Incident Fracture^a^

	Height (cm)					
	<155	155−159	160−164	165−169	170+	All women
Characteristics at study baseline	*n* = 132,636	*n* = 116,428	*n* = 240,168	*n* = 185,015	*n* = 121,834	*n* = 796,081
Mean age, years (SD)	59.6 (5.0)	59.5 (4.9)	59.5 (4.9)	59.3 (4.9)	59.2 (4.8)	59.4 (4.9)
Mean height at recruitment, cm (SD)	152.4 (3.2)	157.5 (0.0)	161.5 (1.3)	166.4 (1.2)	172.6 (3.0)	162.2 (6.6)
Mean measured height, cm (SD)	152.7 (3.6)	156.5 (2.3)	160.4 (2.9)	164.9 (2.8)	170.1 (3.8)	161.5 (6.3)
Mean weight, kg (SD)	63.3 (11.5)	65.9 (11.6)	68.2 (11.7)	70.9 (11.9)	75.1 (12.7)	68.7 (12.4)
Mean BMI, kg/m^2^ (SD)	27.1 (5.0)	26.5 (4.6)	26.1 (4.5)	25.5 (4.3)	25.2 (4.2)	26.0 (4.5)
Mean alcohol, g/d (SD)	6.6 (14.3)	6.9 (14.1)	7.3 (14.0)	7.7 (13.9)	7.8 (14.2)	7.3 (14.1)
Mean no. of children at recruitment (SD)	2.2 (1.2)	2.1 (1.2)	2.1 (1.2)	2.1 (1.2)	2.0 (1.2)	2.1 (1.2)
						
Prior fracture (%)	8.3	8.1	8.3	8.5	9.2	8.5
Prior osteoporosis (%)	6.9	5.7	5.3	4.9	4.7	5.5
One or more fall in the past year (%)^b^	20.1	19.5	19.6	20.2	20.7	20.0
Diabetes (%)	4.6	4.1	3.6	3.2	3.3	3.7
Current smoker (%)	13.7	13.1	11.8	11.4	11.6	12.2
Socioeconomic status: lowest fifth (%)	21.6	18.6	16.0	14.3	14.1	16.6
Ever users of menopausal hormones (%)	53.7	53.6	54.2	54.7	54.3	54.2
Nulliparous (%)	10.3	10.1	10.7	11.8	13.6	11.2
No strenuous activity (%)	63.3	61.8	59.9	58.3	58.7	60.2
No physical activity (%)	30.4	29.5	29.4	29.7	30.1	29.8
Follow‐up for fracture incidence						
Woman‐years of follow‐up (in thousands)	1111	982	2029	1565	1031	6717
All incident fractures (*n*)	4246	3802	8235	6870	5278	28,431
Arm						
Humerus (*n*)	494	418	870	732	522	3036
Radius and ulna (without wrist) (*n*)	249	242	465	452	367	1775
Wrist (*n*)	1471	1259	2879	2313	1762	9684
Leg						
Neck of femur (*n*)	730	671	1598	1462	1273	5734
Femur (not neck) (*n*)	135	125	170	167	116	713
Patella (*n*)	85	101	176	163	124	649
Tibia and fibula (without ankle) (*n*)	300	260	514	424	313	1811
Ankle (*n*)	865	771	1631	1306	950	5523
Other						
Clavicle, spine and rib (*n*)	340	299	654	499	382	2174

^a^ Women with missing values were excluded when calculating the means or percentages for that given variable.

^b^ Among 470,066 women who reported on falls 4 years after study baseline; the percent that reported one or more falls in the previous year.

**Figure 1:**
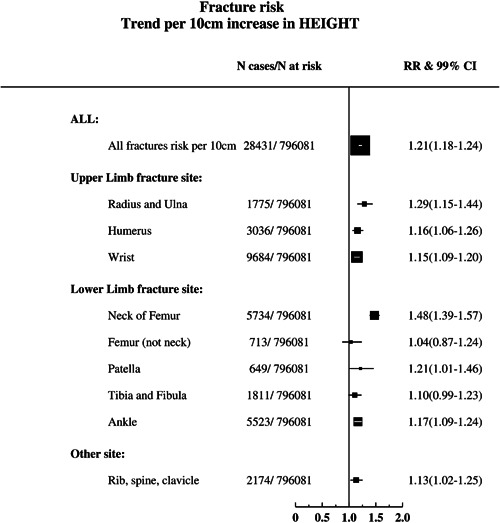
Relative risks and 99% CIs per 10‐cm increase in measured height for incident hospital admission for fracture at various sites and total fractures in postmenopausal women. Results were adjusted for age, socioeconomic status, BMI, strenuous activity, smoking, alcohol consumption, use of hormone‐replacement therapy, diabetes diagnosis, history of prior fracture and history of osteoporosis, and stratified by study region. Mean values of measured height within self‐reported categories were used for trend calculation.

Taller height was associated with an increased risk of fracture in minimally adjusted models (Supplemental Fig. S1), with estimates not much changed by adjustment for other health and lifestyle factors (Fig. 1). In the adjusted model, the relative risk of any fracture increased by 21% (RR = 1.21; 99% CI 1.18–1.24) for each 10‐cm increase in height. Increasing height was significantly associated with an increased risk of hospital admission for an incident radius and/or ulna (RR = 1.29; 99% CI 1.15–1.44), humerus (RR = 1.16; 99% CI 1.06–1.26), wrist (RR = 1.15; 99% CI 1.09–1.20), neck of femur (RR = 1.48; 99% CI 1.39–1.57), patella (RR = 1.21; 99% CI 1.01–1.46), or ankle (RR = 1.17; 99% CI 1.09–1.24) fracture. Neck of femur fracture was associated with the greatest increase in risk per 10‐cm increase in height, significantly greater than for any of the other individual fracture sites studied (*p*_heterogeneity_ < 0.001). Fracture risk also increased with height for the other sites (RR = 1.15 per 10 cm, 99% CI 1.12–1.18); however, the evidence for a possible difference in risk between the sites was very weak (*p*_heterogeneity_ = 0.03). Although risk of fractures of the femur (not neck) (RR = 1.04; 99% CI 0.87–1.24) and the tibia and/fibula (RR = 1.10; 99% CI 0.99–1.23) were not significantly associated with height, risk at these sites did not differ significantly from the overall pattern of an increase in risk.

**Figure 2:**
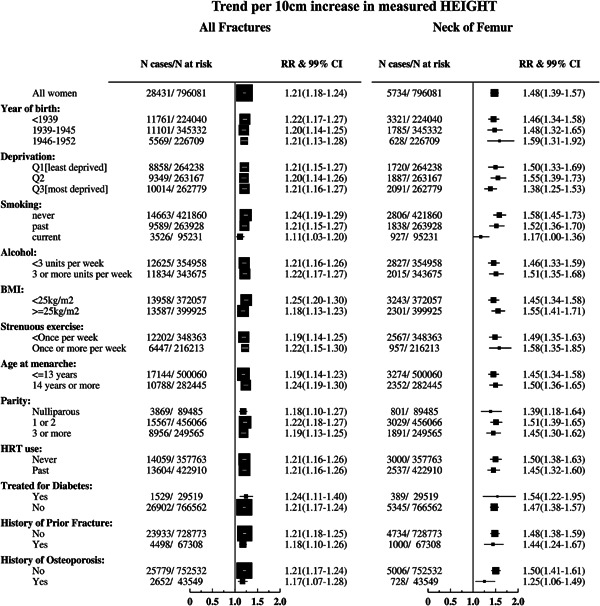
Relative risks and 99% CIs per 10‐cm increase in measured height for all incident fractures at various sites, by various characteristics, in postmenopausal women, mutually adjusted and stratified on region. Mean values of measured height within self‐reported categories were used for trend calculation.

There was little evidence, after accounting for multiple testing, of differences between subgroups of women in the relative risks of any fracture per 10‐cm increase in height according to BMI (*p* = 0.01), birth cohort (*p* = 0.8), age at menarche (*p* = 0.05), deprivation levels (*p* = 0.9), smoking status (*p* = 0.3), alcohol consumption (*p* = 0.7), strenuous physical activity (*p* = 0.4), parity (*p* = 0.4), hormone therapy use (*p* = 1.0), diabetic status (*p* = 0.5), history of prior fracture (*p* = 0.3), or history of osteoporosis (*p* = 0.4) (Fig. 2). Nor was there any evidence of differences in the relative risks of neck of femur fracture per 10‐cm increase in height according to the characteristics listed above (*p* ≥ 0.01) (Fig. 2).

Relative risks did not change substantially in sensitivity analyses, after excluding the first 2 years of follow‐up or excluding women with missing adjustment variables (Supplemental Figs. S2 and S3).

## Discussion

In this prospective cohort study of 796,081 postmenopausal women, taller women were at an overall 21% increased risk of hospital admission for incident fracture at nine major sites per 10‐cm increase in height. Fracture of the neck of femur showed a significantly greater proportionate increase in fracture risk with increasing height (48% per 10 cm) than fractures at other sites. The increase in risk for other fracture sites did not differ greatly from each other.

Significant relationships between increasing height and risk of all fracture sites combined^32^ and all nonvertebral fractures combined^18^, ^33^ have been reported in postmenopausal women. For neck of femur fracture, most larger studies, but not all, have shown an increased risk of neck of femur fracture with increasing height,^10^, ^11^, ^12^, ^34^ similar to our findings. Research into the association of height with fractures at other sites in postmenopausal women has been limited, and, where available, has shown conflicting results. For example, results from two studies suggested no association between wrist fracture and height,^14^, ^34^ whereas we found a small increase with height. A study of postmenopausal women showing an increase in the risk of radial fracture alone with height^13^ was in line with our results indicating an increased risk of forearm fracture among taller women. In contrast to our findings of a small increase in ankle fracture risk with height, no significant increase was reported in four previous studies.^14^, ^16^, ^17^, ^34^ Although we found a small increase in humerus fracture risk with height, height was not a risk factor for humerus fracture among postmenopausal women in two prior studies.^13^, ^15^ One study of postmenopausal women^13^ showed a decrease in risk of vertebral fracture, whereas three other prospective studies did not.^18^, ^19^, ^34^ A prospective study of various fracture sites in postmenopausal women found clavicle fracture to be negatively associated with an increase in height, yet they found no association with height for rib, lower leg, and upper leg fracture.^34^ It is likely that some of the previous studies, often based on small sample sizes, did not have sufficient power to detect the associations between height and fracture. Many previous studies have also not adjusted their findings for well‐known risk factors for fracture such as osteoporosis.

A combination of mechanisms may influence the relationships between height and fracture risk. Some of the association may be biomechanical. During a fall, taller and/or heavier women experience greater forces on impact when compared with shorter and/or lighter women.^1^, ^2^ Hayes and colleagues^3^ demonstrated that a modest, 50‐joule increase in potential energy, which could be accounted for by a 9‐cm increase in fall height, resulted in a 50% increase in the odds of neck of femur fracture (odds ratio = 1.5, 95% CI 1.2–1.9). The length of the moment arm is important in determining the likelihood of fracture after a fall; the longer the moment arm, the less force required for a resultant fracture.^4^ Height has been associated with other geometric features of bones, such as neck of femur axis length,^5^, ^6^ which have in turn been linked to a greater incidence of neck of femur fractures.^4^, ^7^ A cadaver study showed that the ability of the tibia to withstand a torsional force is strongly correlated with the polar moment of inertia, demonstrating the importance of bone size when assessing risk of ankle fracture specifically.^35^ The bone type (eg, long, flat, irregular, sesamoid, or flat) and hence the bone structure involved in the fracture may also play an important role in the height‐fracture relationship. Volumetric bone mineral density has been shown to significantly decrease with each standard deviation increase in height for the distal tibia, distal fibula, and distal radius.^8^ That is, to maintain the competing requirements of strength and lightness in long bones, taller women tend to develop wider bones with more porous and relatively thinner cortices.^8^ This may predispose these taller women to fracture, especially with the increased rate of bone remodeling after menopause.^8^, ^9^

The strengths of this study include the large sample size, prospective nature, and objective recording of incident fractures through NHS hospital admissions. Hospital admissions data did not include fractures presenting at emergency departments or elsewhere, which did not lead to a day‐case or overnight admission; therefore, less serious fractures may not have been captured. Another limitation of this study is the lack of a measure of bone mineral density, which may have provided further insight into the associations between height and fracture at different sites. Although height was self‐reported, we used the mean measured height within each baseline category of self‐reported height to minimize any effects of measurement error. Additionally, correlations between self‐reported height in a subsample of women with measured height were high, thus substantial biases associated with measurement error are unlikely.

Taller women were at an overall increased risk of hospital admission for fracture, especially so for fracture of the neck of femur. Height appears to be an independent risk factor for fractures, with little evidence of substantial confounding or effect modification by other factors for any of the fracture sites. Although height is not a modifiable risk factor, among individuals already at higher risk for fracture, such as those suffering from osteoporosis or those with low adiposity or sarcopenia, it may be an important predictor of fracture risk. Height also varies between different populations and could be one determinant of population differences in fracture incidence.^36^ Height has increased by around 10 cm, on average, in Europeans during the 20th century.^36^, ^37^, ^38^ This may have increased overall fracture risk by around 20% and hip fracture risk by around 50% during this period, if the changes in height have been independent of changes in other risk factors.

## Disclosures

VB is a non‐executive director of the Medicines and Healthcare Products Regulatory Agency. All other authors state that they have no conflicts of interest.

## Supporting information

Supporting Figures 1‐3.
