# Supplementary material for: Relationship of Height to Site‐Specific Fracture Risk in Postmenopausal Women
Source: J Bone Miner Res. 2015 Dec 6;31(4):725–31. doi: 10.1002/jbmr.2742 (PMC4832288; doi:10.1002/jbmr.2742)
Supplement: Supplementary file 1 — Supporting Figures 1‐3. [file JBMR-31-725-s001.docx]

**SUPPLEMENTARY INFORMATION**

**Supplemental Figure 1: Minimally adjusted relative risks and 99% CIs per 10cm increase in measured height for incident hospital admission for fracture at various sites and total fractures in post-menopausal women ^a b^**


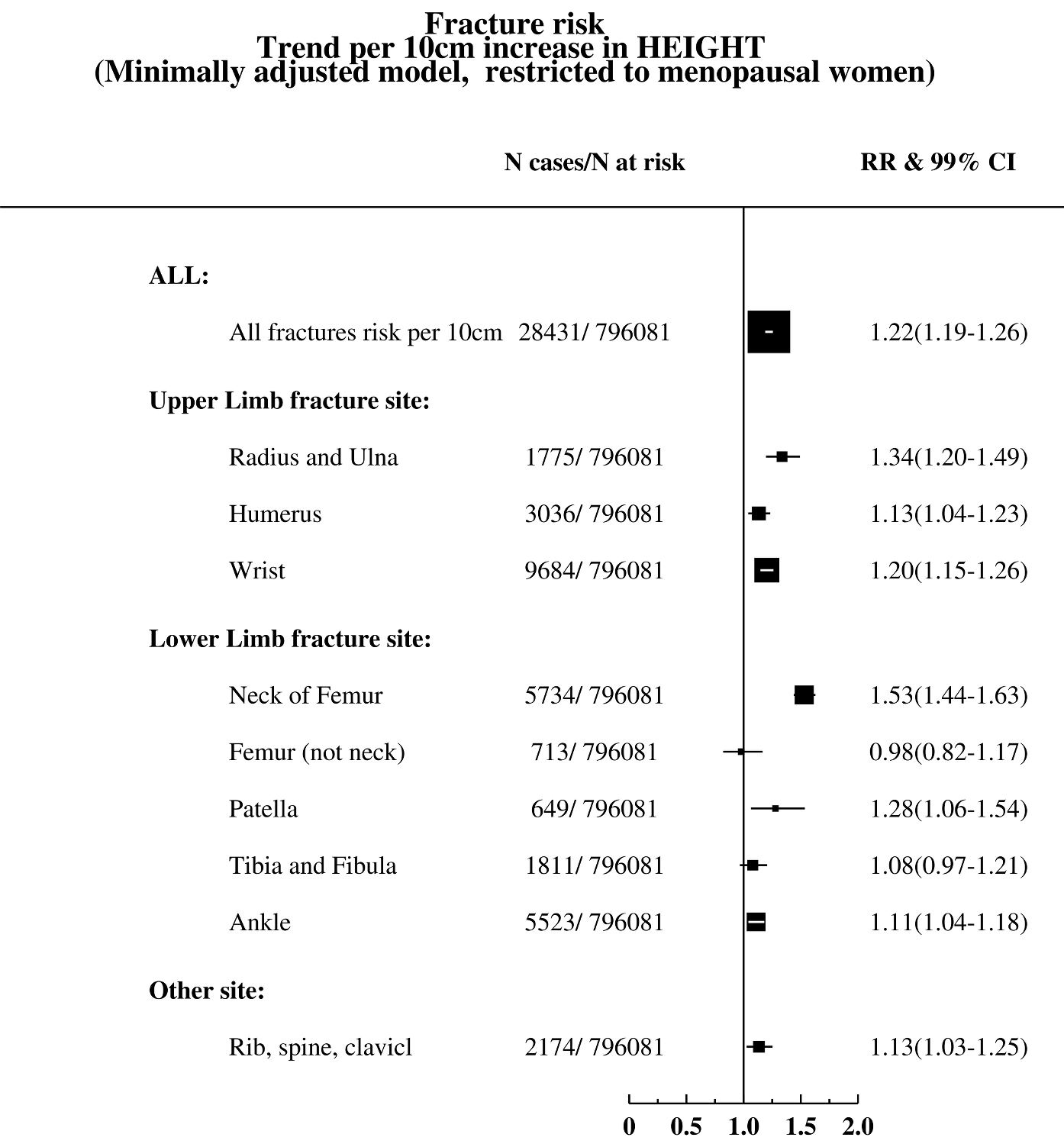


^a^ Minimally adjusted for age and stratified by study region

^b^ Mean values of measured height within self-reported categories used for trend calculation

Supplemental Figure 2: Relative risks and 99% CIs per 10cm increase in measured height for incident fractures at various sites, excluding the first two years of follow-up ^a b^


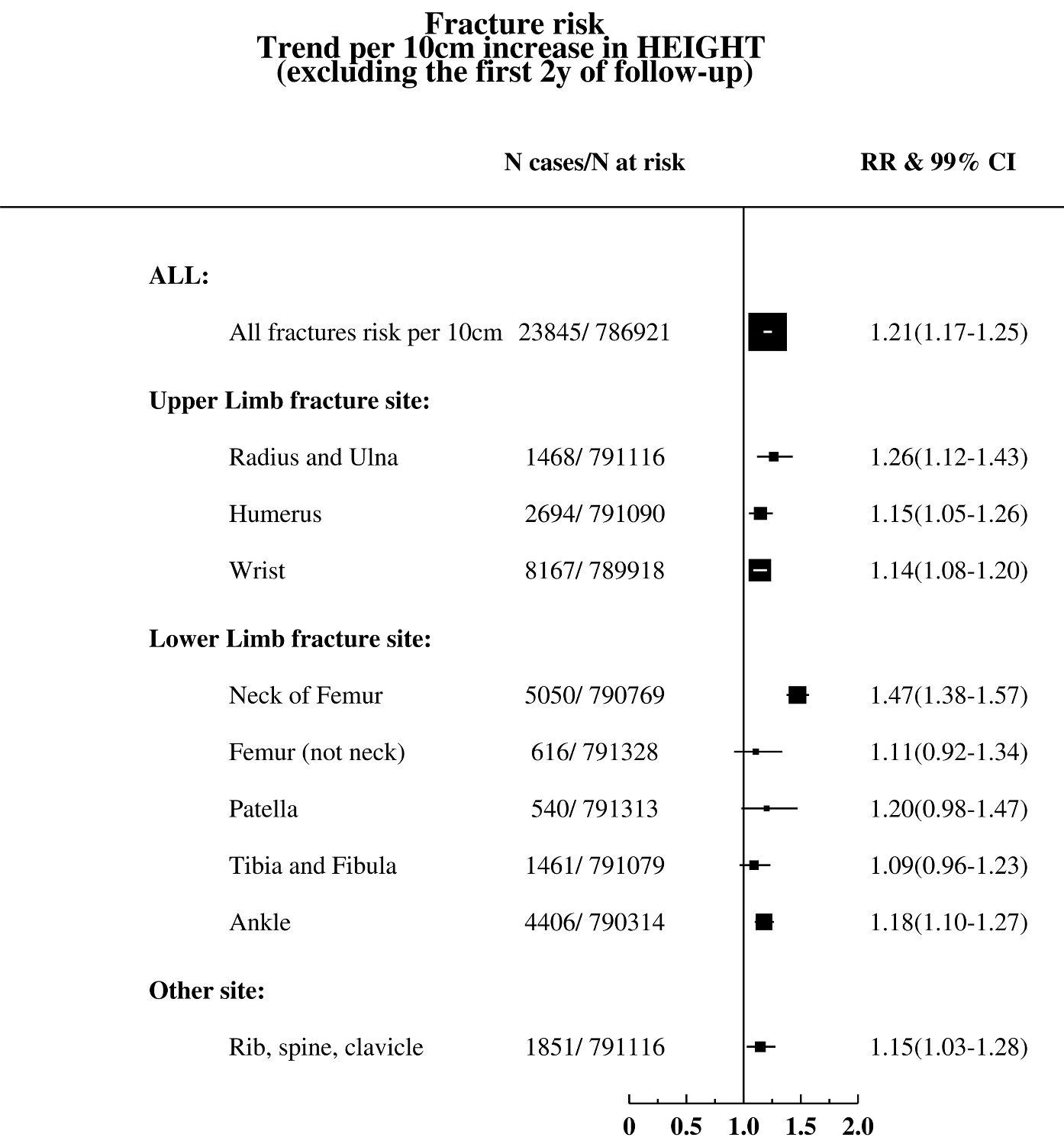


^a^ Adjusted for age, socio-economic status, BMI, strenuous activity, smoking, alcohol consumption, use of HRT, diabetes diagnosis, history of prior fracture and history of osteoporosis, and stratified by study region

^b^ Mean values of measured height within self-reported categories used for trend calculation

Supplemental Figure 3: Relative risks and 99% CIs per 10cm increase in measured height for incident fractures at various sites, restricted to women without missing data ^a b^


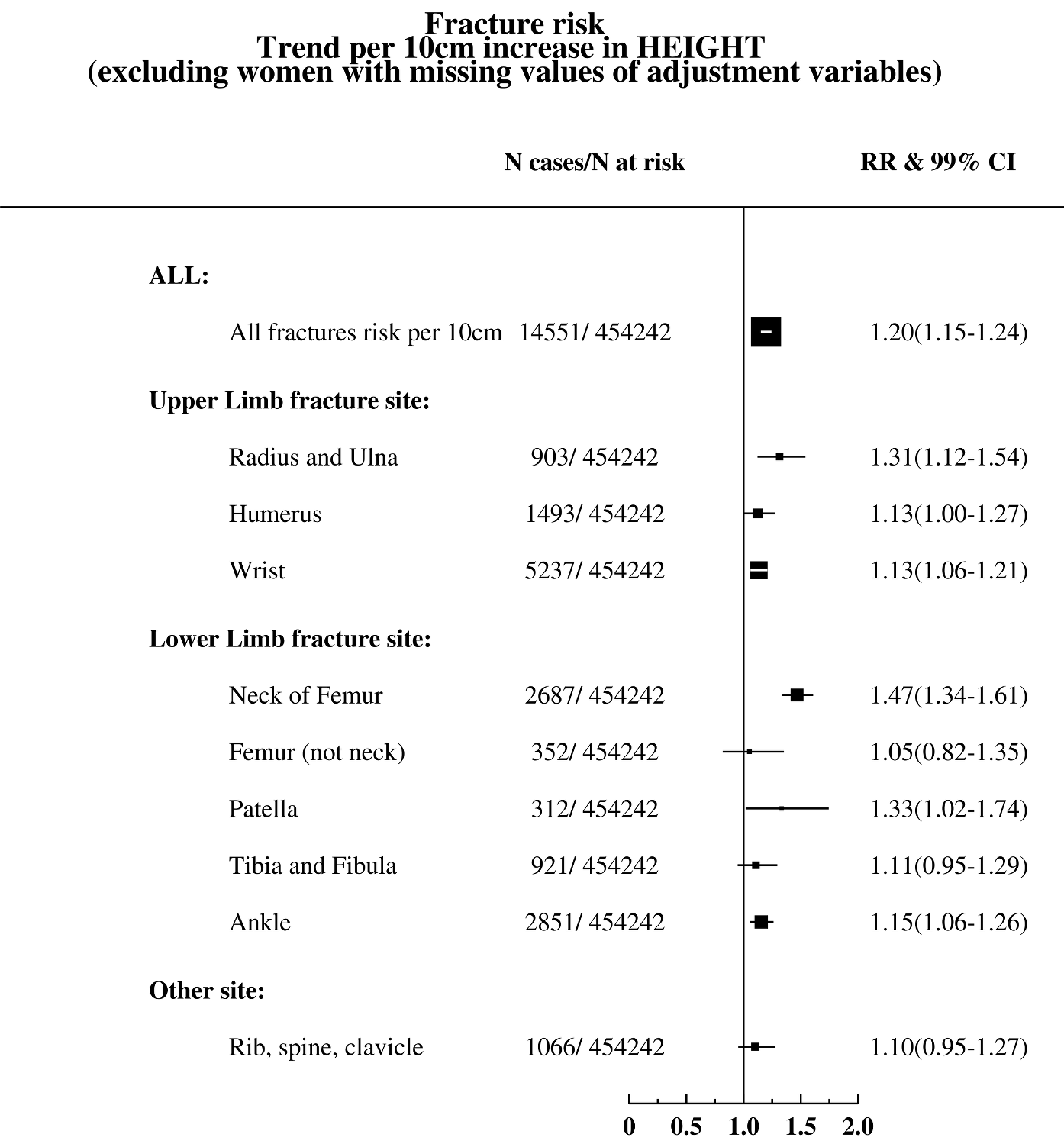


^a^ Adjusted for age, socio-economic status, BMI, strenuous activity, smoking, alcohol consumption, use of HRT, diabetes diagnosis, history of prior fracture and history of osteoporosis, and stratified by study region

^b^ Mean values of measured height within self-reported categories used for trend calculation
